# FGF4 Independent Derivation of Trophoblast Stem Cells from the Common Vole

**DOI:** 10.1371/journal.pone.0007161

**Published:** 2009-09-24

**Authors:** Elena V. Grigor'eva, Alexander I. Shevchenko, Nina A. Mazurok, Eugeny A. Elisaphenko, Antonina I. Zhelezova, Alexander G. Shilov, Pavel A. Dyban, Andrey P. Dyban, Ekaterina M. Noniashvili, Sergey Ya. Slobodyanyuk, Tatyana B. Nesterova, Neil Brockdorff, Suren M. Zakian

**Affiliations:** 1 Institute of Cytology and Genetics, Russian Academy of Sciences, Siberian Department, Novosibirsk, Russia; 2 Institute of Experimental Medicine, Russian Academy of Medical Sciences, St. Petersburg, Russia; 3 Department of Biochemistry, University of Oxford, Oxford, United Kingdom; Universidade Federal do Rio de Janeiro (UFRJ), Instituto de Biofísica da UFRJ, Brazil

## Abstract

The derivation of stable multipotent trophoblast stem (TS) cell lines from preimplantation, and early postimplantation mouse embryos has been reported previously. FGF4, and its receptor FGFR2, have been identified as embryonic signaling factors responsible for the maintenance of the undifferentiated state of multipotent TS cells. Here we report the derivation of stable TS-like cell lines from the vole *M. rossiaemeridionalis*, in the absence of FGF4 and heparin. Vole TS-like cells are similar to murine TS cells with respect to their morphology, transcription factor gene expression and differentiation *in vitro* into derivatives of the trophectoderm lineage, and with respect to their ability to invade and erode host tissues, forming haemorrhagic tumours after subcutaneous injection into *nude* mice. Moreover, vole TS-like cells carry an inactive paternal X chromosome, indicating that they have undergone imprinted X inactivation, which is characteristic of the trophoblast lineage. Our results indicate that an alternative signaling pathway may be responsible for the establishment and stable proliferation of vole TS-like cells.

## Introduction

**Figure 1:**
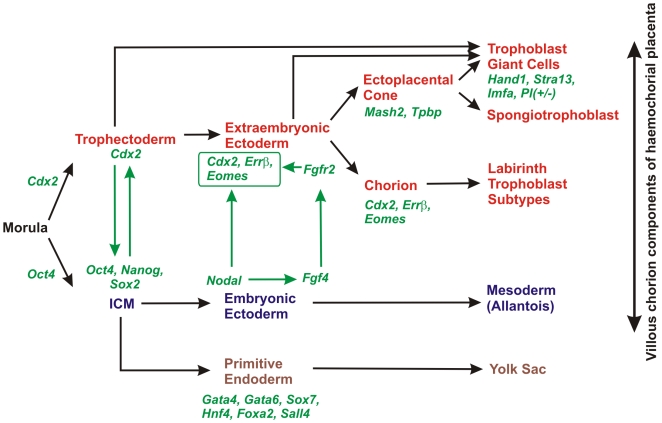
Transcription factor network regulating the development of ICM- and trophectoderm-derived tissues in mouse. From [2] with modifications.

The trophectoderm is the first specialised cell lineage formed by the developing embryo in mammals. It surrounds the blastocoel and undifferentiated inner cell mass (ICM) that will give rise to the embryo proper and to the extraembryonic endoderm tissues. After implantation, multipotent cells of the trophectoderm undergo differentiation into specialised cell types that form the developing placenta. Mural trophectoderm surrounding the blastocoel forms primary giant cells that invade the uterus and lead to embryo implantation. The ICM-contacting polar trophectoderm continues to proliferate and forms the extraembryonic ectoderm (ExE) and, later, the ectoplacental cone [1], which serves as one of the sources of the chorionic plate, and of secondary giant cells (Fig. 1) [2].

It has been shown that the fate of trophectoderm cells is determined by their proximity to the ICM and depends on the signals from the ICM and, later, from the epiblast. Disruption of ICM signaling leads to trophoblast differentiation into giant cells or other terminally differentiated placental cell types [1], [3]. Cells in close proximity to the ICM/epiblast remain diploid and retain the ability to proliferate and give rise to trophoblast stem (TS) cells when cultured *in vitro* [4].

It has been established that the fibroblast growth factor 4 (FGF4) signaling pathway is the main signaling pathway required for proliferation and self-renewal of TS cells and polar trophectoderm/extraembryonic ectoderm [4]-[7]. FGF4 is highly expressed in the ICM/epiblast, and in the presence of heparin it binds to, and activates the membrane-associated *Fgf* receptor 2, expressed by the trophoblast lineage [8], [9]. Embryos mutant for *Fgf4*, or *Fgfr2* show peri-implantation lethality, caused by defects in endoderm derivatives as well as placental defects [10], [11], which indicates the importance of the FGF4 signaling pathway for proliferation of the trophoblast lineage.

The addition of exogenous FGF4 and heparin to culture medium allows the derivation of murine multipotent TS cells when cells are grown in the presence of embryonic fibroblast conditioned medium, or on feeders [4], [12]. These multipotent TS cells exhibit a gene expression profile characteristic of trophectoderm and extraembryonic ectoderm, and are capable of differentiating into various types of trophoblast lineage derivatives both *in vitro*, and *in vivo*, when injected into blastocysts [4]. The removal of heparin, FGF4 or the conditioned medium causes a decrease in cell proliferation and differentiation into giant trophoblast cells. These data confirm the importance of the FGF4/FGFR2 signaling pathway for the maintenance of TS-specific gene expression that, in turn, is necessary for the derivation and maintenance of murine multipotent TS cell lines [4], [13], [14]. However, the requirement for conditioned medium or embryonic fibroblasts indicates that other factors must play a role in the maintenance of the trophoblast lineage.

TS cells represent an excellent experimental model for studying trophoblast development *in vitro* and for analysing the molecular mechanisms of placental development and embryo implantation. Studies conducted on mutant mice bearing various placental defects have indicated that signaling pathways between embryo and trophoblast play a crucial role in successful placental morphogenesis (for review see [13]). In this respect, studies of signaling pathways that control the self-renewal of stem cells, and the interaction between different types of stem cells in other species, are of particular interest. They provide a novel perspective on the complex signaling network regulating trophoblast development and can facilitate our understanding of the molecular processes governing placental morphogenesis in other species, including humans.

It is known that different sets of transcription factors that trigger species-specific signaling pathways are required to maintain the self-renewal of embryonic stem (ES) cells in different mammalian species. For instance, basic FGF is essential for the derivation and maintenance of human ES cells [15], while the pleiotropic cytokine LIF (leukemia inhibitory factor) is required to activate the JAK/STAT3 signaling pathway responsible for the maintenance of mouse ES cell pluripotency [16]–[18]. These examples demonstrate that the success of stem cell derivation for any particular species may depend on the choice of the growth factors and/or culturing conditions.

Here we report the derivation of TS-like cell lines for another rodent species, the common vole *M. rossiaemeridionalis,* from 3.5 days postcoitum (dpc) blastocysts in the absence of FGF4. Surprisingly, the presence of vole LIF in the culture medium was essential for the derivation of these lines, but was not necessary for their maintenance. In mice, LIF is involved in maintaining the pluripotency of murine ES cells through activation of the JAK/STAT3 signaling pathway and is not required for TS cell derivation or maintenance. Our data indicate the existence of an FGF4-independent signaling pathway that controls the establishment and self-renewal of vole TS-like cells.

## Results

### Isolation of *M. rossiaemeridionalis* stem cell lines

Previously, we attempted to derive vole ES cells from early blastocysts [19] but we were only able to isolate ES-like cells, with limited multipotency. One of the likely causes of this failure was the use of heterologous (murine) LIF, a secreted glycoprotein involved in the regulation of the growth and differentiation of different cell types. To overcome this problem, we engineered and purified recombinant species-specific vole LIF protein (see Text S1 and Fig. S1).

**Figure 2:**
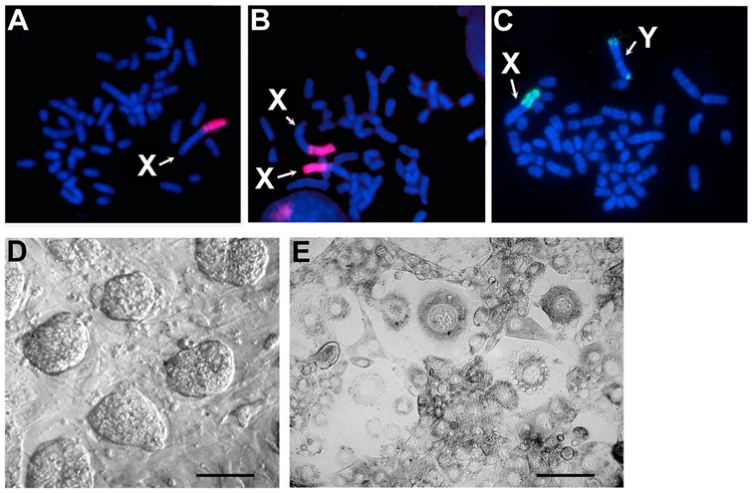
Characterisation of *M. rossiaemeridionalis* TS-like cell lines. Fluorescent *in situ* hybridization (FISH) of vole sex chromosome specific heterochromatin repeats MS4 (red and green) on metaphase chromosomes obtained from three TS-like cell lines with the sex chromosome compositions: X0 (A), XX (B), and XY (C). Chromosomes are counterstained with DAPI. Morphology of undifferentiated vole TS-like cell colonies grown on a feeder layer of embryonic fibroblasts (D) and giant trophoblast cells obtained by spontaneous differentiation of TS-like cells at the 28^th^ passage (E). The scale bar represents 200 µm in (D and E).

Twelve 3.5 dpc blastocysts were plated on a feeder layer of inactivated vole embryonic fibroblasts in the presence of vole LIF protein. After three to five days in culture the ICM outgrowth was dissected out and mechanically dissociated. Each dissociated ICM gave rise to one to 42 primary stem cell-like colonies. The growth rate and extent of spontaneous differentiation of the colonies varied, but the majority differentiated after two to three passages. Colonies from three ICMs maintained a high proliferation index and had morphology typical of mouse undifferentiated TS cells. These gave rise to three stable, independent diploid cell lines with the sex chromosome compositions: XO (R1), XX (R2), and XY (R3) (Fig. 2A to C).

The colonies had a flat, epithelial morphology with visible nucleoli structures (Fig. 2D), and a protuberance of intensively proliferating cells was formed on the periphery of the colonies. These cells are characterised by a high nuclear/cytoplasmic ratio and rapid cell growth (doubling of the colony size in less than 24 hrs), which are features typical of stem cells. The cell lines were maintained for at least 70 passages over a period of nine months, without essential changes in their morphology.

The cell lines we obtained were capable of proliferating stably on the vole feeder layer, in the absence of LIF, without changes in morphology (see Table 1). Mouse embryonic fibroblasts, alone and without LIF, were also able to maintain the derived vole cells in an undifferentiated state. However, LIF alone (either vole or mouse), without embryonic feeder cells, was not sufficient to keep cells undifferentiated, and differentiation was observed as early as the second passage. Differentiation of cells, growing on a gelatinized surface without feeders, could be prevented by cultivating them in 70% conditioned medium collected from inactivated vole, or mouse fibroblasts supplemented with vole, or mouse LIF. Cells retained their stem cell morphology for at least 35 passages under these conditions. The withdrawal of feeders/conditioned medium induced cell differentiation into trophectoderm derivatives, predominately trophoblast giant cells (Fig. 2E), which were detected by analysis of cell morphology and expression of genes specific for differentiated mouse TS cells (see below).

**Table 1:** Specific conditions for derivation and maintenance of vole TS-like cells.

	Conditions	Vole LIF	Mouse LIF	w/o LIF
Derivation conditions	Vole feeder	**√**	**X**	nd
	Mouse feeder	nd	**X**	nd
Maintenance conditions	Vole feeder	**√**	**√**	**√**
	Mouse feeder	**√**	**√**	**√**
	Gelatin	**X**	**X**	**X**
	Gelatin+vole condition medium	**√**	**√**	**√**
	Gelatin+mouse condition medium	**√**	**√**	**√**

Taken together, it is clear that certain unidentified factors, secreted by embryonic fibroblasts, are required for maintaining proliferation of the derived vole cell lines, whereas the presence of vole LIF is a crucial factor only during the initial stages of vole TS-like cells derivation.

### Analysis of stem cell-specific markers

**Figure 3:**
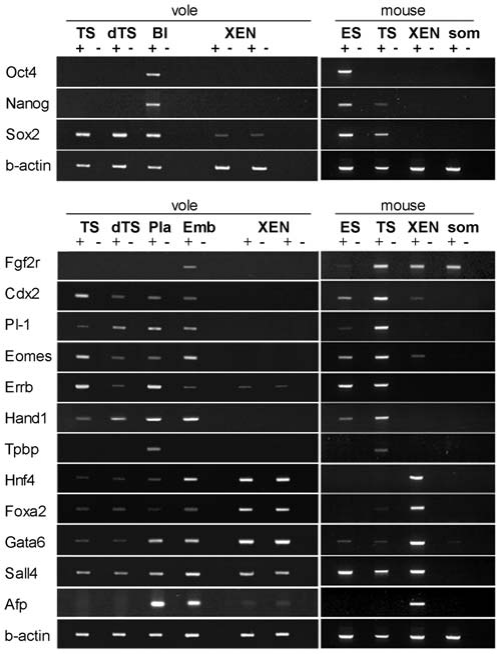
RT-PCR analysis of transcription factors expressed in vole TS-like cells. Transcription factors specific for mouse germ layers and expressed in three TS-like cell lineages: embryonic (ES), extraembryonic ectoderm (TS) and extraembryonic endoderm (XEN). (Bl) 4.5 dpc vole blastocyst; (Pla) 12.5 dpc vole placenta; (Emb) vole embryos at 7.5 dpc; (som) mouse somatic cells.

The unusual behaviour and properties of the derived vole cell lines prompted us to analyse the expression of the stem cell-specific transcription factors *Oct4*, *Nanog* and *Sox2*, in both undifferentiated and differentiated cells (Fig. 3). The expression of *Sox2* was detected in undifferentiated, as well as differentiated vole TS-like cells. While *Oct4* and *Nanog* typical of ES-cells were expressed in epiblast of 4.5 dpc vole blastocysts, they were not detected in the derived cells (Fig. 3). This pattern of expression was reminiscent of mouse TS, rather than ES cells, therefore we expanded the panel to include TS and extraembryonic endoderm (XEN) stem cell-specific markers [20] (Fig. 3).

We found that transcription factors characteristic of the trophectoderm, such as *Cdx2, Eomes* and *Errβ*, were highly expressed in the vole cells, thus supporting the hypothesis that they are derived from the trophoblast. In addition, we detected the presence of *Hand1*, known to promote the differentiation of giant trophoblast cells and to be essential for placental development [21], in both undifferentiated and differentiated cells. It is known that *Hand1* is highly expressed in both differentiated and undifferentiated mouse TS cells [4].

Surprisingly, *Fgfr2* expression was not detected in vole TS-like cells (Fig. 3). The *Fgfr2* gene encodes a surface receptor for FGF4 and is required for maintaining the unlimited proliferation of mouse TS cells [4], [11]. The reason for the absence of *Fgfr2* gene expression in vole cells is unknown at present.

*Placental lactogene* (*Pl*-1) is known to function in the development of the placenta [22]. Accordingly, expression of this gene was not detected in undifferentiated vole TS-like cells, but it was activated in differentiated giant trophoblast-like cells (dTS, Fig. 3).

*Tpbp* expression, specific for the spongioblast and ectoplacental cone [23]–[26], was not detected in the cell lineages analysed, although the expression of this gene was detected in vole placenta.

Expression of the extraembryonic endoderm markers *Hnf4, Foxa2, Gata6* and *Afp* was barely detectable in vole cells, similar to mouse TS cells, however these markers were readily detectable in vole XEN stem cells (Fig. 3 and [27]).

Our expression analysis of lineage-specific genetic markers demonstrates that only genes of the trophoblast lineage are expressed in the established vole cell lines and their differentiated derivatives.

### Subcutaneous injection of vole TS-like cells causes haemorrhagic tumour formation

**Figure 4:**
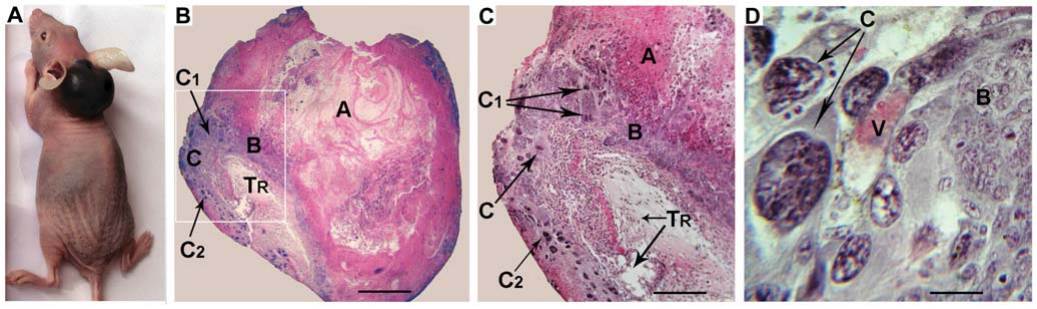
Tumour formation caused by subcutaneous injection of *M. rossiaemeridionalis* TS-like cells into a *nude* mouse. (A) External view of the tumour. (B to D) Histological sections of the haematoma. Staining by hematoxylin-eosin. (B) Cross-section through the central part of the haematoma 35 days after injection. (C) Magnified view of the boxed fragment of the tumour from (B); histological section of the haematoma across a blood vessel (D). (A) necrosis zone, (B) proliferating cells, (C) giant cells, (C_1_) differentiating giant cells, (C_2_) degenerating giant cells, (T_R_) recipient tissues, (V) blood vessel. The scale bar represents 1000 µm in (B), 500 µm in (C) and 10 µm in (D).

In order to analyse the invasive properties of vole TS-like cells, we injected undifferentiated R1 cells subcutaneously into *nude* mice. In four *nude* mice, one to two weeks after injection, the injected cells caused the development of tumours that were morphologically reminiscent of haemorrhagic tumours (Fig. 4A). Histological analysis of the tumour sections revealed that each tumour was comprised of an inner blood-filled capsule. PCR amplification of vole-specific gene products from the inner capsule confirmed that it was derived from the injected vole cells (data not shown). Moreover, culturing the capsule tissue under stem cell conditions gave rise to colonies that were identical in morphology to the injected cells (data not shown). This result indicated the presence of a pool of undifferentiated cells within the tumour capsule.

Detailed histological analysis also showed that the tumour contained both viable and dying trophoblast cells. Tumours were surrounded by a layer of actively proliferating cells, comprised of giant trophoblast cells at different stages of differentiation (Fig. 4B to D). At the periphery of the actively proliferating cell layer, aggregates of mainly uninucleate differentiated giant cells were detected. These regions were permeated by capillary networks. No embryonic germ layer derivatives were detected in the tumours.

Therefore, our data demonstrate that the subcutaneous injection of vole TS-like cells causes cellular invasion into the host blood vessels and erosion of blood vessel walls, resulting in the development of large haemorrhagic tumours. Similar data have been reported previously for the subcutaneous injection of mouse TS cells and human choriocarcinoma cells [28], [29].

### X chromosome inactivation is imprinted in vole TS-like cells

Mouse undifferentiated XX ES cells carry two active X chromosomes, while undifferentiated TS cells exhibit imprinted inactivation of the paternally inherited X chromosome [12]. *Xist*, a key player in the X inactivation process [30], and its antisense partner *Tsix* [31] are reliable markers of X inactivation as their transcriptional status reflects the activity of the X chromosomes. Both *Xist* and *Tsix* are transcribed at low levels from the active X chromosomes in undifferentiated XX and XY ES cells, while high levels of *Xist* transcript alone are detected in differentiated XX cells, which contain one inactive X chromosome [31]–[33].

**Figure 5:**
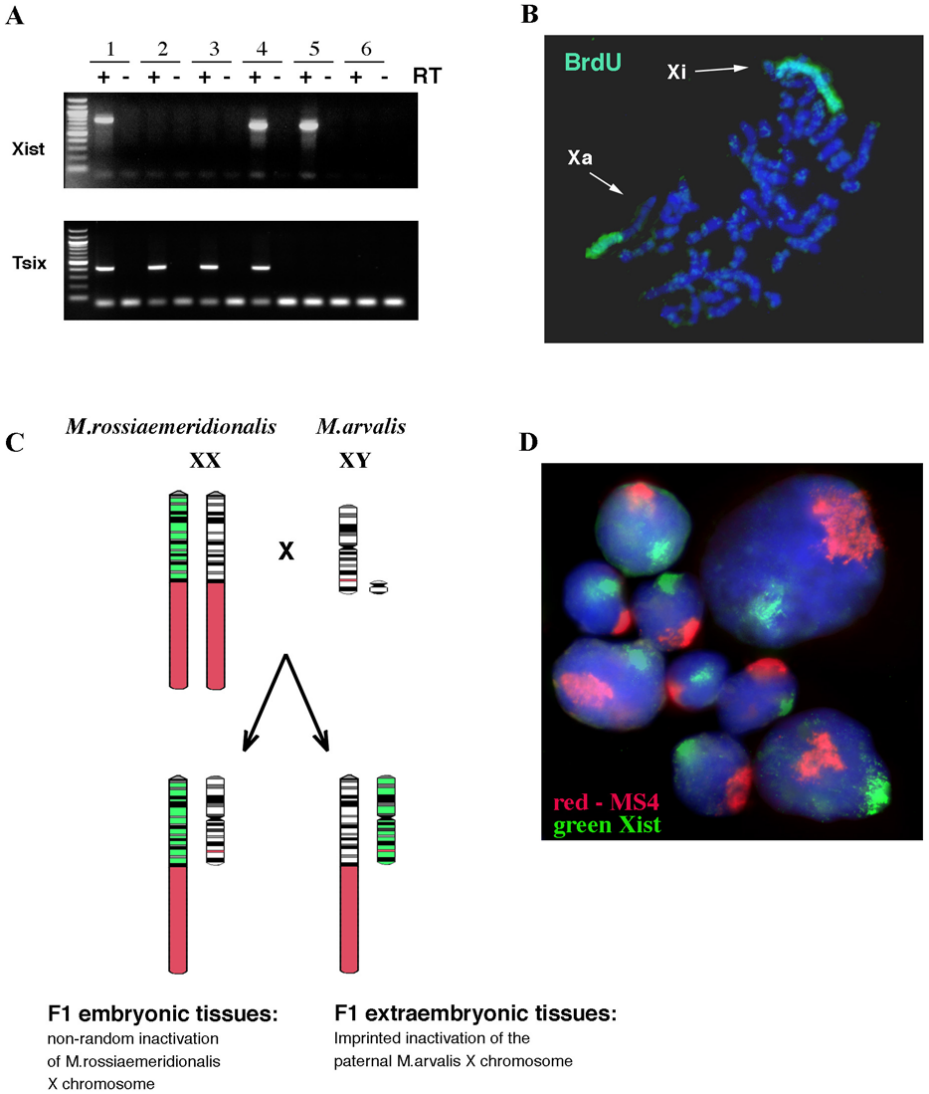
Imprinted inactivation of the X chromosome in vole TS-like cells. (A) RT-PCR analysis of *Xist* and *Tsix* expression in vole TS-like cells: vole placenta (1); X0 undifferentiated (2) and differentiated cells (3); XX undifferentiated (4) and differentiated cells (5); mouse feeders (6). (B) *M. rossiaemeridionalis* XX TS-like cells exhibit late replication of one of the X chromosomes, as detected by a FITC-coupled anti-BrdU antibody (green). Note that the entire inactive chromosome (Xi) is stained, while only the constitutive heterochromatin region is stained on the active (Xa) chromosome. Chromosomes are counterstained with DAPI. (C) Schematic showing the pattern of X chromosome inactivation in embryonic and extraembryonic tissues of *M. rossiaemeridionalis* x *M. arvalis* vole hybrids. *Xist* RNA on the inactive X chromosome (green), the *M. rossiaemeridionalis* heterochromatin block (red). (D) RNA-DNA FISH analysis of the X inactivation pattern in TS-like cells derived from an *M. rossiaemeridionalis* x *M. arvalis* hybrid embryo. *Xist* RNA is detected with FITC (green) and the *M. rossiaemeridionalis* X chromosome heterochromatin specific repeat MS4, is detected with Texas Red (red).

To establish the X chromosome inactivation status in vole TS-like cells, we performed strand-specific RT-PCR analysis of vole *Xist* and *Tsix* genes. As shown in Fig. 5A (lane 4), *Xist* expression is high in undifferentiated XX cells, where it is presumably associated with the inactive X, whereas *Tsix*, presumably associated with the active X chromosome, shows weaker expression. When vole TS-like cells are maintained in differentiated conditions for 12 to 14 days, they lose *Tsix* expression completely, most likely due to repression of the *Tsix* promoter on the active X chromosome (Fig. 5A, lane 5). Low levels of *Tsix* transcript were found in vole XO undifferentiated stem cells but no *Xist* transcripts were detected. This observation supports the suggestion that *Tsix* is expressed from the active X chromosome in undifferentiated vole TS-like cells (Fig. 5A, lanes 2 and 3). The fact that undifferentiated vole TS-like cells have a high level of *Xist* expression indicates that inactivation of one of the parental X chromosomes has already occurred.

To verify the inactive state of the X chromosome, we analysed the X chromosome replication patterns in undifferentiated stem cells from *M. rossiaemeridionalis*, which carry large blocks of heterochromatin on the distal part of the X chromosome [34]. It is well documented that the inactive X chromosome generally replicates later in S phase than the active X chromosome and the autosomes [35]–[37]. Using anti-BrdU antibodies to detect BrdU incorporated in late S phase of the cell cycle, we found that one X chromosome was entirely late replicating, while the other showed only the heterochromatic part as late replicating, and the euchromatic part as early replicating (more than 50 metaphases were examined; Fig. 5B). These data support the hypothesis that undifferentiated vole TS-like cells carry an inactive X chromosome.

However, it is still unclear whether the derived vole undifferentiated cells have undergone random, or imprinted X inactivation. If the cells are differentiated derivatives of ES-like cells from the ICM, we should expect random X inactivation. If the cells are TS-like, then X inactivation should be imprinted, with the paternal X chromosome chosen to be inactive. To discriminate between these two possibilities, we took advantage of the distinct morphology of the X chromosomes between two vole species, *M. arvalis* and *M. rossiaemeridionalis* (Fig. 5C). Previously we showed that these two species can produce viable offspring, and female hybrids demonstrate an extremely skewed X inactivation pattern, with the *M. rossiaemeridionalis* X chromosome being chosen preferentially as the inactive one in somatic tissues [38], [39]. We derived an F1 hybrid stem cell line, where the maternal X chromosome was inherited from *M. rossiaemeridionalis* and the paternal X came from *M. arvalis* (Fig. 5C). The heterochromatin-specific repeat MS4 [40] was used as a marker of the maternal *M. rossiaemeridionalis* X, thereby allowing us to establish unequivocally if *Xist* was being transcribed from the maternal, or paternal X chromosome. If the *Xist* domain localises together with the MS4 domain, then the X inactivation pattern is somatic-type, in which the *M. rossiaemeridionalis* X is preferentially inactivated. However, if the two domains are detected separately, then this points to imprinted X inactivation. As shown in Fig. 5D, the MS4 domain always localises separately from the *Xist* domain. This clearly indicates that the derived vole cells have undergone imprinted X inactivation and confirms that they belong to the TS cells.

## Discussion

### Vole stem cells belong to the trophectoderm lineage

We derived stable TS-like cell lines from vole *M. rossiaemeridionalis* blastocysts. The derived colonies of the vole cells by visual microscopic analysis reveal a flat, epithelial morphology with tight, intercellular contacts, a high nuclear/cytoplasmic ratio, they maintain multipotency after prolonged culturing *in vitro* and differentiate exclusively into derivatives of trophoblast lineages. The morphology of the cell lines obtained is similar to that of ES-like cells in primates [41], human [42], sheep, pig [43], [44], rabbit [45] and murine TS cells [4], [14], [46].

We detected the presence of the transcription factors *Cdx2* and *Eomes*, which are critical for the determination and maintenance of the murine trophectoderm lineage [47], [48], in the derived vole cell lines. We also showed that the transcription factors *Errβ*, as well as *Sox2*, which are necessary for the maintenance of the multipotency of murine TS cells [13], [49], [50], were present in the vole undifferentiated lines. Differentiation of these cells caused changes in cell morphology and activation of the transcription factor *Pl-*1, a marker of murine TS cell differentiation towards trophoblast giant cells [4]. Importantly, we did not detect the expression of the transcription factors *Oct4* and *Nanog*, which are crucial for self-renewal and maintenance of the pluripotent state of ES cells [51]–[54] in the analysed vole cells. Therefore, the pattern of transcription factor expression indicates that the derived vole stem cells belong to the TS, rather than the ES cell lineage, in spite of their derivation without FGF4, and in the presence of LIF.

One of the intrinsic properties of trophoblast stem cells is their ability to form fast growing haemorrhagic tumours following subcutaneous injection into *nude* mice. This ability was demonstrated previously for human choriocarcinoma cells [28] as well as for mouse undifferentiated TS cells [29] that invaded and eroded host blood vessels and formed lacunas filled with blood. Trophoblast cells exercise this ability *in vivo* during the formation of the haemochorial placenta, when giant trophoblast cells invade maternal tissues during implantation, to establish a continuous blood supply to the developing embryo [55]. Similar to mouse trophoblast cells and human choriocarcinoma cells, subcutaneous injection of the derived vole cells into *nude* mice leads to the formation of haemorrhagic tumours. Histological examination of the tumours revealed the presence of invading giant trophoblast cells, originating from the injected donor vole cells, in the mouse tissues surrounding the blood vessels.

One of the characteristics of mouse XX TS cells is that they carry a transcriptionally silent paternal X chromosome, similar to the cells of the trophectoderm germ layer from which they are derived [12]. Here we show conclusively that vole stem cells have undergone imprinted X inactivation, a further indication of their TS identity.

In summary, the evidence provided in this study indicates that our established vole cell lines demonstrate properties of the trophoblast lineage and should be regarded as vole TS-like cells.

### Activation of an alternative signaling pathway in vole TS-like cells

It has been established that FGF4 is a component of the embryonic signaling pathway required for the maintenance of the multipotent state of the trophoblast lineage [4], [13]. It functions through the binding and activation of the surface receptor *Fgfr2*, which is expressed by trophoblast cells. Mice with targeted mutations of either *Fgf4*, or its receptor *Fgfr2*, have similar severe peri-implantation phenotypes, signifying the importance of this signaling pathway. The lethality is due to defects in the endoderm derivatives, as well as in trophoblast cell proliferation [10], [11]. To date, the data indicate that activation of the FGF4/FGFR2 signaling pathway is important for maintaining cellular proliferation during normal embryonic development.

Previously it was shown that FGF4 and heparin are two crucial factors in the establishment and proliferation of murine TS cells [4], [13], [14]. Withdrawal of these factors from the culture medium causes cell growth arrest and differentiation into giant trophoblast cells. Remarkably, we show in this study, that vole TS-like cells can be established in the absence of FGF4 and heparin. Moreover, vole TS-like cells do not express the FGF4 receptor gene *Fgfr2*, suggesting that an alternative signaling pathway, or pathways are activated in these cells.

Our characterisation of the derived vole cell lines confirms their trophoblast lineage identity. Therefore our data indicate that FGF4 is not absolutely essential for TS cell establishment, at least in some species. Vole TS-like cells are also able to proliferate and maintain their multipotency after prolonged passaging *in vitro*, in the absence of Fgf4/Fgfr2 signaling. However, we should note that the derivation efficiency of stable TS-like cell lines in voles, under these conditions, is quite low whereas FGF4-assisted TS cell derivation was reported to be very efficient in mice [4], [12].

The requirement for embryonic fibroblast-conditioned medium, or embryonic feeder cells, in addition to exogenous FGF4, for the derivation and maintenance of murine TS cells led Tanaka *et al*. (1998) to suggest that other unidentified factors might be involved in maintaining TS cells. The *Estrrb* (≡*Errβ*) gene, which encodes the orphan nuclear receptor ERR-β, was suggested as a potential candidate for an FGF4-independent signaling pathway, since embryos mutant for this gene die at around 10.5 dpc from severe deficiency of a diploid trophoblast [13], [49]. High levels of *Errβ* expression in vole TS-like cells corroborate this hypothesis. Our data also indicate that other factors are required, either independently of the FGF4 and/or ERR-β pathways, or as upstream regulators of the *Errβ* gene, to maintain vole TS-like cells in a proliferating undifferentiated state.

Recent studies have shed more light on the mechanisms involved in the regulation of TS cell fate in murine extraembryonic ectoderm, and on the identity of factors essential for maintenance of TS cell proliferation [56], [57]. It was shown that a member of the transforming growth factor (TGF) beta superfamily, Nodal, is required to sustain the continuous expression of *Fgf4*. Further to this, Nodal acts in parallel with FGF4, to maintain normal expression of *Errβ, Eomes* and *Cdx2*, thereby preventing differentiation of TS cells [56]. The important role played by the TGFβ superfamily was further emphasised in a study showing that conditioned medium may be substituted by the TGFβ superfamily factors, activin A, and TGFβ [57]. It is therefore plausible that the TGFβ signaling pathway predominates in vole TS-like cells and that it acts directly to support the expression of *Errβ, Eomes* and *Cdx2*, without any cooperation with the FGF4 signaling pathway. The involvement of other, as yet unidentified factors is also possible. We should note that the factors controlling the maintenance of vole TS-like cell proliferation must be highly conserved, as mouse feeders were capable of maintaining the proliferation of undifferentiated vole TS-like cells.

While the nature of the vole factor(s) remains unknown, the fact that species-specific LIF is required for vole TS-like cell derivation and early maintenance confirms the importance of LIF in launching this signaling pathway. LIF belongs to a large family of pleiotropic cytokines and demonstrates a wide range of diverse activities affecting gene regulation, cell proliferation and differentiation. Contrary to its action in somatic cells, LIF maintains the self-renewal and pluripotency of ES cells through the activation of the canonical JAK/STAT3 pathway. Interestingly, it was reported that *c-myc*, rather than *Oct4* is a candidate target gene activated by Stat3 [58], although the linking factor between *Stat3* and *Oct4* has not yet been identified. It is obvious that the presence of LIF is not sufficient to sustain *Oct4* expression and to derive vole ES cells, therefore it is likely that vole LIF triggers some other signaling pathway that leads to the derivation of vole TS-like cells.

At present we do not know why the FGF4/FGFR2 signaling pathway is not required for vole TS-like cell derivation/maintenance, or whether other exogenous factors are required to trigger it; however the existence of an FGF4-independent signaling pathway in this process opens up exciting opportunities for studying the signaling networks that regulate trophoblast development. Such studies could also assist in the establishment of TS cell models for other species, including human, for which attempts to derive TS cell lines have so far failed.

In this report we describe the derivation of stable TS-like cell lines from vole *M. rossiaemeridionalis* blastocysts. Vole TS-like cells are similar to murine TS cells in their morphology and molecular and physiological properties, however the requirement of different conditions for their derivation and maintenance implies the existence of an FGF4-independent signaling pathway that might be triggered by LIF. The presence of high concentrations of LIF is dispensable after the derivation stage, and conditioned medium/embryonic feeders alone are sufficient to keep the cells undifferentiated. Vole TS-like cells may provide a useful tool for studying alternative signaling pathways and for the identification of new factors responsible for trophoblast development.

## Materials and Methods

### Derivation and culture of vole TS-like cells

The East European common vole *M. rossiaemeridionalis* (see [59] for its systematic position within the order Rodentia) was used for stem cell derivation. Animals trapped in their natural habitats were bred in the vivarium of the Institute of Cytology and Genetics (Novosibirsk, Russia). The study was done with permission of the Ethical Committee of the Institute of Cytology and Genetics, Novosibirsk. Timed matings were set up and the morning when sperm was detected in the vaginal smear was counted as 0.5 dpc. Morulas and blastocysts (early, middle, and late) were flushed from the oviducts and uteri at 3.5 dpc. The zona pellucida was removed by incubation in acidified Tyrodes solution for 1 to 1.5 min (see [60] for details). The embryos were then placed on a layer of mitomycin C-inactivated embryonic fibroblasts (feeder) in four well plates (individual one embryo per well or two to three embryos per well) in stem cell medium (see below). Vole embryos at 12.5 to 14.5 dpc were used as a feeder source. The inactivated fibroblasts were plated on gelatinized four well plates at a concentration of 1.5×10^5^ cells per well. Cells were maintained at 37°C in an atmosphere of 5% CO_2_.

After three to five days, the ICM was separated mechanically from the trophoblast outgrowth in phosphate buffered saline (PBS) or in culture medium. The polar trophectoderm has not been removed during dissection of the ICM. The ICM was dissociated into small pieces with fine glass capillaries, and cells were re-plated on a new four well plate with fresh feeders. Stem cell-like colonies were selected from a variety of primary cellular outgrowths on the basis of their morphology, i.e. actively growing compact colonies, composed of small tightly packed cells without visible cell boundaries and with a high nuclear/cytoplasmic ratio. Depending on the cell growth rate, the colonies were removed from the plate and trypsinised (0.025% trypsin, 0.037% EDTA Na_2_, 2% chicken serum) into small cell clumps after five to ten days.

TS-like cells were maintained in 1∶1 mixture of Dulbecco's modified Eagle's medium (DMEM) and Ham's F-12 medium supplemented with 15% fetal calf serum (FCS, Autogen Bioclear), vole LIF (see Text S1 and Fig. S1), 1 mM L-glutamine, 1% nonessential amino acids, 0.1 mM 2-mercaptoethanol, penicillin (50 units/ml), streptomycin (50 µg/ml) and nucleosides (0.01 mM guanosine, 0.03 mM adenosine, 0.01 mM cytidine, 0.01 mM uridine and 0.01 mM thymidine, Sigma). All reagents were from Invitrogen, unless otherwise is stated.

### RNA preparation and RT-PCR

RNA was isolated with RNA-Bee reagent (Biogenesis). All samples were treated with DNAse I to ensure they were free from DNA contamination (Turbo DNA-free, Ambion). SuperScriptIII (Invitrogen) was used for cDNA synthesis primed from random decamers. The primers SDx3: 5′cccagtgctggtgagctattcc, and btdf: 5′gaaacacctccaccgcactacact were used for the strand-specific cDNA synthesis of *Xist* and *Tsix* transcripts, respectively. PCR primers and conditions are given in the Table 2. Vole PCR products were sequenced to confirm their homology to the respective mouse genes (See Table 2 for GenBank Acc. numbers).

**Table 2:** Primers and PCR conditions for vole lineage-specific genes.

Gene	Primer sequences	Primer names	product size, bp	Conc., MgCl_2_, mM	Tm, °C	GenBank Acc. for vole transcripts
*β-actin*	gacggggtcacccacactgt gagtacttgcgctcaggaggag	β-actin-1 β-actin-2	523	3	56	
*Oct4*	ccaagctgctgaagcagaaga tttgaatgcatgggagagcccag	OCT4-2F OCT-5R	631	4	53	EF030115
*Eomes*	attgtccctggaggtcggta gaaggtcgggtcagggtaat	Eom8F Eom6R	316	3	56	EU285608
*Hand1*	ccccatgctccacgaaccc aactcccttttccgcttgct	Hand1F1 Hand1R4	467	3	58	EU285606
*Fgf2r*	tcttgttcttcaggggacgattc atgcttcccactatttactcctctg	FGFR2F2 FGFR2R3	242	3	60	DQ517964
*Errβ*	agctgcggctccttcatcaag cttgtacttctggcggcctcc	ERRB1F ERRB4R	543	2	63	DQ517967
*Cdx2*	ccaccatgtacgtgagctacc gactgagcgctgtccaagtt	Cdx2F1 Cdx2R2	184	2	60	DQ517966
*Pl-1*	tccagagaatcgagaggaagt acaactcggcacctcaagac	Csh1F1 Csh1R2	383	3	60	DQ517968
*Tpbp*	aaacagccactgtgccattg gtaaggttttattagtgtgaacat	Tnbp3F Tnbp7R	214	2	60	DQ517965
*Foxa2*	ccctacgccaatatgaactccatg gttctgccggtagaaaggga	Foxa2F1 Foxa2R2	220	3	60	EU285609
*Hnf4*	ggtcaagctacgaggacagc agaagatgatggctttgagg	Hnf4F1 Hnf4R5	460	3	60	EU285605
*Sox2*	tccatgaccagctcgcagacctac ccctcccaattcccttgtttctct	Sox2F Sox2R2	392	3	60	EU285607
*Nanog*	tccataacttcggggagga tcacagagtagttcaggaata	NanogF NanogR	156	4	52	EU285611
*Afp*	acatcgaggagagccaggca cctgagcttggcacagatcc	Afp11F Afp10R	413	3	60	EU285612
*Gata6*	caccatcatcaccacccgacctac cagggccagagcacaccaagaatc	Gata6F Gata6R	788	3	60	EU285604
*Sall4*	tcaccaacgccgtcatgttacagc ggtgggctgtgctcggataaatgt	Sall4F Sall4R	604	3	60	EU285610

### RNA FISH and late replication analysis

RNA FISH was performed as described [61], [62]. To analyse asynchronous X-chromosome replication, the cell culture was maintained in a medium supplemented with BrdU (final concentration 25 µg/ml) for six hours prior to cell harvesting. After the standard hypotonic treatment and cell fixation, the slides were incubated in 4N HCl, following by BrdU detection with anti-BrdU antibody coupled with FITC (Abcam) at a 1∶5 dilution for 30 min.

### Cytogenetic analysis

Hypotonic treatment and cell fixation, as well as differential staining of the chromosomes, were performed as described [59]. The length of the hypotonic treatment was 20–30 min.

### Isolation and analysis of tumours

A cell suspension (8 to 9×10^6^ cells in 150 µl of medium without supplements) was injected subcutaneously into the neck area between the ears of *nude* mice. After one to two weeks the animals were culled and the haematomas were analysed. The walls of the haematoma capsules were fixed in Bouin solution [63] and tissue sections were analysed histologically.

## Supporting Information

Text S1 Cloning and expression of vole leukemia inhibitory factor (LIF) ′
(0.02 MB DOC)

Figure S1 LIF amino acid sequences in three species: human, mouse, vole (M. rossiaemeridionalis). The signal peptide sequences (aa 1 to 14) are underlined. (hu) human, (mo) mouse, (vo) vole. Positions with interspecies amino acid substitutions are given in grey.
(0.31 MB TIF)
