# Supplementary material for: FGF4 Independent Derivation of Trophoblast Stem Cells from the Common Vole
Source: PLoS One. 2009 Sep 24;4(9):e7161. doi: 10.1371/journal.pone.0007161 (PMC2744875; doi:10.1371/journal.pone.0007161)
Supplement: Text S1 — Cloning and expression of vole leukemia inhibitory factor (LIF) ′ (0.02 MB DOC) [file pone.0007161.s001.doc]

**Text S1**

*Cloning and expression of vole leukemia inhibitory factor (LIF)*

Vole LIF cDNA was amplified by RT-PCR. Total RNA was isolated from *M. rossiaemeridionalis* embryonic fibroblast cells. Conserved 5’ and 3’ untranslated regions of mouse and human LIF mRNA were used to design primers to amplify the vole LIF homologue (Lfor1: 5’tccccatttgagcatgaac and Lrev1: 5’gacctcctctctagaaggcctg). The nucleotide sequence of the vole PCR fragment was obtained with an automated sequenator (Applied Biosystems) using the primers above. To produce recombinant vole LIF protein, we performed another round of PCR amplification with the primers SapLF: 5'ggtggttgctcttccaacgttctgcactggaaacac and BamLR: 5'ggtggtggatccttagaaggcctgggccaccat. The PCR product was digested with SapI and BamHI and cloned into the expression vector pTWIN1 (New England BioLabs). The *E. coli* ER2566 strain carrying the Т7 RNA polymerase gene was used for transformation of the construct into bacterial cells. The homogeneity of the LIF preparation was checked by electrophoresis.

Vector NTI software was used for translation of the DNA sequence into the amino acid sequence, and for calculation of the ratio of the molar extinction of LIF. Comparative analysis of 613 bp of coding sequence of the LIF transcript, between vole and mouse, revealed 66 nucleotide substitutions; of these 35 substitutions were found at the third codon position, while 31 were found at the first and second positions. Twenty seven nucleotide substitutions caused amino acid substitutions, of which 12 are responsible for the alteration of either hydrophobic properties or charge, or bending of the polypeptide chain. Comparison of LIF amino acid sequences in human, mouse, and vole is illustrated in Fig. S1.
