# Supplementary figures and images for: FGF4 Independent Derivation of Trophoblast Stem Cells from the Common Vole
Source: PLoS One. 2009 Sep 24;4(9):e7161. doi: 10.1371/journal.pone.0007161 (PMC2744875; doi:10.1371/journal.pone.0007161)

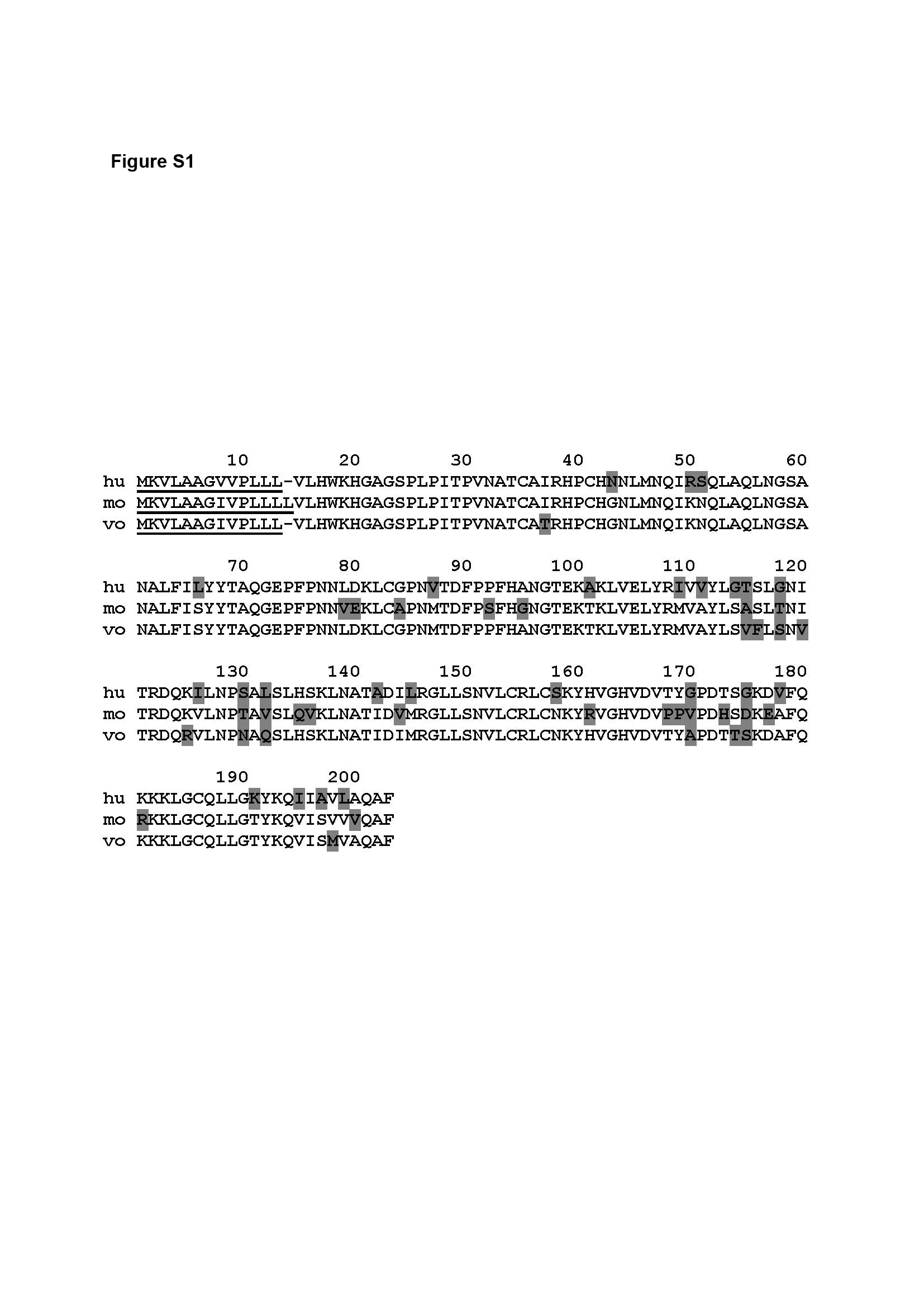

Supplement: Figure S1 — LIF amino acid sequences in three species: human, mouse, vole (M. rossiaemeridionalis). The signal peptide sequences (aa 1 to 14) are underlined. (hu) human, (mo) mouse, (vo) vole. Positions with interspecies amino acid substitutions are given in grey. (0.31 MB TIF) [file pone.0007161.s002.tif]
